# Synergistic Effect of Essential Oils and Rhamnolipid on *Xanthomonas citri* Subsp. *citri*

**DOI:** 10.3390/microorganisms13051153

**Published:** 2025-05-17

**Authors:** Maria Olimpia Pereira Sereia, Eduarda Araujo dos Santos, Lucas Prado Leite, Raphael Culim Neves, Vítor Rodrigues Marin, Henrique Ferreira, Jonas Contiero, Daiane Cristina Sass

**Affiliations:** 1 Department of General and Applied Biology, Institute of Biosciences, São Paulo State University (UNESP), Rio Claro 13506-900, Brazil; olimpia.sereia@unesp.br (M.O.P.S.); eduarda.araujo@unesp.br (E.A.d.S.); vitor.marin@unesp.br (V.R.M.); henrique.ferreira@unesp.br (H.F.); 2 Institute of Research on Bioenergy, São Paulo State University (UNESP), Rio Claro 13506-900, Brazil; lp.leite@unesp.br (L.P.L.); raphael.culim@unesp.br (R.C.N.); jonas.contiero@unesp.br (J.C.)

**Keywords:** citrus canker, phytochemicals, biosurfactants, bioactive combination, sustainable agriculture

## Abstract

Citrus canker, caused by *Xanthomonas citri* subsp. *citri*, is a devastating disease that affects citrus production and trade worldwide. Traditional control methods, based on copper compounds, are effective but pose environmental and health risks due to their toxicity and potential for bioaccumulation. This study evaluates the synergistic potential of essential oils (EOs) and rhamnolipids as sustainable alternatives for disease management. Four EOS (citronella, palmarosa, geranium, and clove) were tested for their antibacterial activity. Citronella EO showed a 90% inhibitory concentration (IC 90) of 0.15% (*v*/*v*) and a minimum bactericidal concentration of 0.25% (*v*/*v*), while the other EOs showed IC 90 and bactericidal activity at 0.06% (*v*/*v*). Rhamnolipids (RHLs), biosurfactants produced by *Pseudomonas aeruginosa*, inhibited *X. citri* at a concentration of 0.3% (*v*/*v*). The combination of citronella EO and RHLs showed a synergistic effect, reducing the inhibitory concentration of citronella by 50% and that of RHLs by more than 90%. In addition, the combined formulation permeabilized more than 80% of bacterial membranes and reduced biofilm formation. In contrast, other oils tested in combination with rhamnolipid showed independent effects. These results indicate that EOs and rhamnolipids represent an environmentally safe strategy for the control of *X. citri* subsp. *citri* that overcomes the limitations of conventional methods while reducing environmental and health impacts.

## 1. Introduction

Citrus canker, caused by the bacterium *Xanthomonas citri* subsp. *citri*, is a disease that severely affects commercially important citrus species, having impacts on fruit production and trade [1,2]. When the plant is infected, severe symptoms, such as leaf loss, premature fruit drop, and severe fruit discoloration, occur. Infection spreads rapidly in warm and humid weather conditions, resulting in defoliation and reduced fruit quality [3]. These effects not only undermine productivity, but also reduce the market value of the crop, resulting in economic losses for growers and the citrus industry as a whole. The ability of *X. citri* subsp. *citri* to form biofilms plays a central role in both its epiphytic and pathogenic forms [4]. Biofilm formation allows bacteria to adhere to and colonize leaf surfaces, establishing a stable niche that precedes the onset of visible disease symptoms [5,6]. In addition, the structured biofilm matrix provides protection against desiccation, ultraviolet radiation, and antimicrobial agents, which together increase the fitness and ability of the pathogen to initiate successful infections [7]. Consequently, biofilms are not only survival strategies, but integral components of the disease cycle, underpinning the epidemiological success of *X. citri* subsp. *citri* in citrus-producing regions worldwide [5].

Traditionally, the control of this disease has relied on copper-based formulations, which, despite their efficacy, remain the main chemical control method for *X. citri* [2,8,9,10,11,12,13] However, the use of these formulations poses serious risks to human health and the environment, as copper is a bioaccumulative heavy metal with mutagenic and carcinogenic effects [14,15,16]. In addition, its application to soil can lead to the death of various organisms and disrupt entire ecosystems [17]. Considering this scenario, it is essential to search for effective and environmentally safe alternatives for the control of citrus canker. In this context, essential oils (EOs) have emerged as promising options due to their low toxicity to mammals and reduced environmental persistence due to their volatility [18]. In addition, they have demonstrated antibacterial activity against several bacterial species. For example, Xiao and coworkes identified 39 essential oils, including citronella, palmarosa, geranium, and clove, with antibacterial activity against *Staphylococcus aureus* in the stationary phase, a stage associated with persistent infections [19]. In the case of the genus *Xanthomonas*, studies also highlight the efficacy of EOs. Another study evaluated the antibacterial activity of essential oils from *Cymbopogon* species, such as citronella and palmarosa, against *X. citri* subsp. *citri*, highlighting their potential as natural alternatives for the disinfection of citrus fruits [20]. Complementarily, Nagy et al. investigated the antibacterial efficacy of essential oils, such as clove and citronella, against *Xanthomonas arboricola* pv. *pruni*, using microdilution and direct bioautographic assays, with clove oil showing particularly high efficacy [21]. These results reinforce the potential of EOs as sustainable tools for the management of diseases caused by pathogenic bacteria, highlighting their viability as natural alternatives for the control of citrus canker and similar diseases.

However, the industrial application of essential oils and their constituents as antimicrobial agents is limited mainly due to their hydrophobicity [22]. Therefore, it is crucial to develop appropriate formulations to allow their use on a commercial scale. In this sense, rhamnolipids, biosurfactants belonging to the class of glycolipids, have shown promise [23]. Synthesized mainly by the bacterium *Pseudomonas aeruginosa*, but also by other microorganisms, such as *Acinetobacter* sp. and *Enterobacter* sp. [19], rhamnolipids exhibit surfactant properties that remain stable over a wide range of pH levels and temperatures [23,24]. They also exhibit antimicrobial activity against fungi and bacteria, including *X. citri* [25,26,27]. In agriculture, rhamnolipids have been recommended for pathogen control in greenhouses as both as antimicrobial agents and as inducers of plant defense responses [26].

Recent studies have shown that, due to their excellent emulsifying properties, rhamnolipids can exhibit synergistic effects when combined with bioactive molecules, thereby enhancing biological activity [27,28]. For example, Haba et al. [28] demonstrated the synergistic effect of emulsions formed by rhamnolipids and four essential oils on *Staphylococcus aureus* and *Candida albicans*. However, to date, there is no information on the use of rhamnolipids as emulsifiers in essential oil formulations aimed at inhibiting *X. citri*.

Therefore, the aim of this study was to investigate the synergistic effect of citronella, palmarosa, geranium, and clove essential oils in combination with rhamnolipids, and to evaluate their respective activity against *X. citri* subsp. *citri.*

## 2. Materials and Methods

### 2.1. Bacterial Strains and Culture Conditions

The *X. citri* subsp. *citri* used in all experiments was the isolate WT306 (IBSBF 1594) obtained from the IBSBF Culture Collection Instituto Biológico (Campinas, São Paulo, Brazil). The bacteria were grown in Nutrient Yeast Glycerol liquid medium (5 g L^−1^ of peptone, 3 g L^−1^ of yeast extract, 2% *v*/*v* of glycerol, and 20 g L^−1^ of bacteriological agar for solid media), at 29 °C ± 1 °C for 24–48 h with shaking at 200 rpm when necessary. The reagents used in the experiments, including resazurin dye, crystal violet, DAPI, and propidium iodide (PI) stains, as well as the Yeast Minimal Medium, were all commercially purchased from Sigma-Aldrich (Darmstadt, Hesse, Germany). The statistical analysis was performed using the GraphPad software Prism v.6 (GraphPad, San Diego, CA, USA).

### 2.2. Essential Oils and Rhamnolipid

The essential oils (EOs) of citronella, palmarosa, geranium, and clove were obtained commercially from the company OSHADI^®^ (lot: 2023-001) (Colombo, Paraná, Brazil), and the rhamnolipids were prepared by the research group of Prof. Dr. Jonas Contiero, as described in the literature [25,29], using *Pseudomonas aeruginosa* LBI 2A1 from the random mutant bank at the Laboratory of Industrial Microbiology, Department of General and Applied Biology, UNESP, Rio Claro, São Paulo State, Brazil.

### 2.3. GC-MS Analysis

To characterize the chemical composition of EOs, all samples were analyzed by gas chromatography-mass spectrometry (GC-MS) using the methodology by Marin et al. [30]. GC-MS was performed using a Shimadzu GCMS-QP2010 ULTRA (Kyoto, Japan) with an Rtx-5MS capillary column (5% phenyl, 95% dimethylpolysiloxane) (Restek, Bellefonte, PA, USA) (30 m × 0.25 mm, 0.25 µm film). Helium was used as the carrier gas (1 mL·min^−1^), with a 1:10 split injection. The injector and interface temperatures were 250 °C and 260 °C, respectively. The oven temperature was programmed from 60 °C (2 min hold) to 200 °C at 4 °C·min^−1^, then to 260 °C at 6 °C·min^−1^ (10 min hold) [30]. The ionization source temperature was 280 °C, using the full-scan acquisition mode (70 eV). Compounds were identified using the National Institute of Standards and Technology (NIST) 11.0 EPA/NIH mass spectral library.

### 2.4. Bacterial Growth Inhibition

The evaluation of the inhibitory activity of *X. citri* subsp. *citri* by essential oils (EOs) and rhamnolipids alone was performed using the Resazurin Microtiter Assay Plate (REMA) technique of Palomino et al. [31]. This method, which indirectly measures cellular respiratory activity, allowed the precise determination of the inhibitory concentrations of the compounds. The tests were performed in 96-well plates, with at least three replicates per compound. Each well received the tested compounds at initial concentrations of 100 µg/mL, reduced by serial dilutions of 1/2. Bacteria were added in the appropriate medium at a concentration of 10^5^ cells/well, in a total volume of 100 µL per well. Controls included: negative (medium without compound), positive (kanamycin), and vehicle control (e.g., DMSO 1%). After preparation, the plates were incubated, followed by the addition of resazurin (0.001%). Fluorescence was measured in the Synergy H1 reader (BioTek, Winooski, VT, EUA) with excitation/emission filters at 530/590 nm. The results are expressed as a percentage of cell growth inhibition, with the data processed using GraphPad Prism software (version 6).

This model was used to determine the minimum inhibitory concentration required to inhibit 90% of cell growth (IC 90) and the bactericidal concentration, the minimum bactericidal concentration (MBC), defined as the lowest concentration of an antimicrobial agent required to eliminate 99.9% of the initial bacterial population [32,33,34]. All experimental procedures were performed in triplicate to ensure reproducibility.

### 2.5. Synergism

The combined antibacterial activity of citronella essential oil and rhamnolipid was evaluated using the checkerboard microdilution assay according to the methodology described by Bellio et al. [35]. Stock solutions of both compounds were prepared at twice the minimum inhibitory concentration required to inhibit 90% of bacterial growth (2 × IC 90). The EO was serially diluted along the rows, while the rhamnolipid was diluted along the columns of a 96-well microplate. The outer columns (1 and 12) were filled only with NYG culture medium to minimize evaporation effects and were excluded from the analysis.

A standardized bacterial suspension of *X. citri* subsp. *citri* was adjusted to an optical density of 0.04 at 600 nm, equivalent to approximately 10^8^ CFU/mL, and then diluted 1:10 in the NYG medium. Each well received 10 µL of the bacterial suspension, resulting in a final concentration of 10^5^ cells/well. The microplates were incubated at 29 ± 1 °C for 16 h. After incubation, resazurin (0.015 µg·mL^−1^ final concentration) was added to each well, and the plates were further incubated for 2 h to assess bacterial viability. Fluorescence readings were performed using a Synergy H1 microplate reader (BioTek). All experiments were performed in triplicate.

The synergistic effect was determined by calculating the Fractional Inhibitory Concentration Index (FICI), defined as the sum of the individual Fractional Inhibitory Concentrations (FICs) of the compounds in the combination [36]. The FICI was calculated according to the following formula:

$$\mathrm{FICI}=\mathrm{FIC}\;A+\mathrm{FIC}\;B=\frac{IC\;90\;A\;combined*}{IC\;90\;A\;alone}+\frac{IC\;90\;B\;combined*}{IC\;90\;B\;alone}$$

* IC 90 A combined: Refers to the concentration of compound A in combination with compound B required to inhibit 90% of bacterial growth.
* IC 90 B combined: Refers to the concentration of compound B combined with compound A required to inhibit 90% of bacterial growth.

The interaction of the combination of two substances was defined as a synergistic effect if the FIC index was ≤0.5, additive if 0.5 < FICI < 1, indifferent if 1 < FICI ≤ 4, and antagonistic if FICI > 4.

The concentrations selected for subsequent analyses were based on the combinations that showed a synergistic effect, as determined by the FICI values. Among the synergistic interactions observed, the combination with the lowest effective concentrations of both compounds was prioritized. This approach is consistent with the primary objective of this study, which is to maximize antibacterial efficacy while minimizing the required concentrations of the active compounds, thereby reducing potential cytotoxicity, environmental impact, and formulation costs.

### 2.6. Cell Membrane Permeabilization

The live and dead microscopy assay was performed according to the methodology described by Zamuner et al. [37] to evaluate the mechanism of action of synergistic combinations against *X. citri* subsp. *citri*. Cells were exposed to the compounds according to the REMA protocol, with 10^5^ cells in 100 µL of culture in 1.5 mL microtubes. After the incubation period, the exposure was interrupted by diluting the samples (1:10) in 0.85% saline solution, followed by centrifugation at 2500× *g* for 5 min and discarding the supernatant. The cells were resuspended in 0.85% saline solution, and 10 µL of the suspension was applied to agarose-coated slides for observation.

DAPI staining was used to detect changes in the organization and distribution of the bacterial nucleoid [37]. DAPI penetrates cells with intact cytoplasmic membranes, whereas propidium iodide stains cells with damaged membranes. Cells with permeabilized membranes were stained red (propidium iodide) while cells with intact membranes were artificially blue-stained (DAPI). Visualizations were performed using an Olympus BX-61 (Bridgewater, NJ, EUA) fluorescence optical microscope, with images captured by an OrcaFlash 2.8 camera Hamamatsu (Bridgewater, NJ, EUA) and processed using CellSens Dimension 1.18 (Olympus) software (Hachioji, Tokyo, Japan).

### 2.7. Effect of Bioactive Combinations on Biofilm Production by X. citri Subsp. citri

The biofilm assay was performed using the method of Malamud et al. [38], to quantify biofilms formed in 96-well plates. *X citri* subsp. *citri* colonies were grown in NYG medium, adjusted to an OD600 of 0.4, and diluted in Yeast Minimal Medium (YMM) with compounds at concentrations of 50 and 25 µg/mL. After incubation at 29 °C for 72 h, the biofilm was stained with crystal violet (CV), solubilized with 70% ethanol, and the absorbance measured at 590 nm. Biofilm formation indices were calculated and the data normalized in JASP software v. 0.14.1 [39].

### 2.8. Time-Kill Assay

The time-kill assay described by Gerits et al. [40] was performed using essential oil (EO) and rhamnolipid at a synergistic combination concentration of 1× IC 90, 0.002% (*v*/*v*) rhamnolipid and 0.075% citronella (*v*/*v*), 2× IC 90, 0.004% (*v*/*v*) rhamnolipid and 0.15% citronella (*v*/*v*), and 4× IC 90, 0.008% (*v*/*v*) rhamnolipid and 0.3% citronella (*v*/*v*). *X. citri* was inoculated in NYG medium at a concentration of 10^8^ CFU/mL and incubated on an orbital shaker at 29 °C for 24 h. After the incubation period, 100 μL aliquots were taken from each sample at different time intervals (0, 1, 2, 4, 6, 24, and 48 h). Serial dilution was then performed and a plate counting technique was used to determine the number of viable cells. A bacterium inoculated in NYG without the addition of bioactives was used as a control. All experiments were performed in triplicate.

### 2.9. Statistical Analysis

The REMA assay for the crude extract was performed in triplicate in three independent experiments, with bacterial growth inhibition measured through fluorescence. Data analysis, including the construction of dose–response curves and the determination of IC 90 values along with their 95% confidence intervals (95% CIs), was performed using GraphPad Prism software. A significance level of *p* < 0.05 was used. To evaluate the inhibitory activity of the fractions, a single-replicate screening was performed. Microscopy results were evaluated using Brown–Forsythe and Welch ANOVA to detect significant differences among the treatment groups (*p* < 0.05). Percentage of permeabilized cells was determined by analyzing 200 cells per treatment in duplicate experiments performed on two different days. Biofilm data were evaluated using the unpaired *t*-test and Welch’s ANOVA to determine significant differences (*p* < 0.05). All experiments were conducted in three independent replicates. Two-way ANOVA analysis was performed to examine statistical differences between treatments and time, at the 95% confidence level. Tukey’s post hoc test (95% confidence interval) was used to examine the relationships between treatments at a single time point and between treatments at all time points.

## 3. Results

### 3.1. Bacterial Growth Inhibition

The inhibitory activity of the isolated EOs and rhamnolipids against *X. citri* subsp. *citri* was evaluated, and the results of these individual evaluations were used to prepare mixtures for inhibitory activity. The results obtained with the REMA assay for essential oils and rhamnolipids are shown in Table 1.

### 3.2. GC-MS Analysis

The four essential oils (EOs) studied, palmarosa (*Cymbopogon martinii),* citronella (*Cymbopogon nardus*), geranium (*Pelargonium roseum*), and clove (*Syzgium aromaticum/Eugenia caryophyllata*), were obtained commercially, and their composition was confirmed by analysis using the gas chromatography-mass spectrometry (GC-MS) technique (Table 2, Table 3, Table 4 and Table 5). The sum of the identified compounds corresponds to 100% of the composition of each oil.

In the palmarosa essential oil, the main constituents were geraniol (71%) and neryl acetate (19.17%). For citronella, geraniol (43%) and citral (E) (13.53%) stood out. Geranium oil presented 21 compounds, the most abundant being citronellol (34.52%), geraniol (Z) (15.73%), and citronellyl formate (11.02%). In clove, the main compounds were chavibetol (63.84%) and eugenol acetate (30.86%).

### 3.3. Synergism

The checkerboard assay was used to evaluate the synergistic effect of rhamnolipid in combination with the four oils in vitro. In order to calculate the Fractional Inhibitory Concentration Index (FICI), only those results were considered that showed an inhibition of bacterial growth greater than 90%. Table 6 shows the results obtained in the experiments using rhamnolipid with citronella, palmarosa, geranium, and clove. According to the results obtained by calculating the FICI, a synergistic effect can be observed between citronella essential oil–rhamnolipid (≤0.5), and for the other oils an additive effect is demonstrated (0.5 < FICI < 1) between the substances tested. All experiments were carried out in triplicate.

### 3.4. Cell Membrane Permeabilization

**Figure 1:**
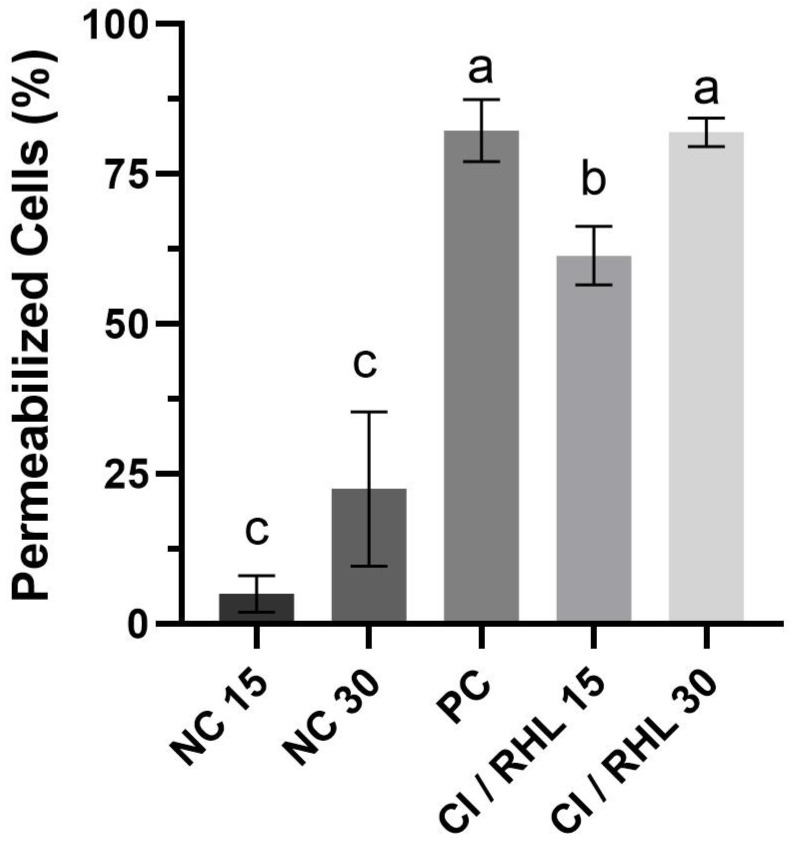
Percentage of *X. citri* subsp. *citri* with permeabilized cell membranes after treatment with citronella essential oil and rhamnolipid. CI: citronella essential oil; RHL: rhamnolipid; NC: negative control; PC: positive control. Error bars represent the standard deviation of the mean. Numbers 15 and 30 represent the duration of exposure to each treatment in minutes. Bars represent the mean of each treatment and whiskers represent the standard deviation. Letters (a), (b), and (c) represent statistically similar groups (*p* value > 0.05) according to the Brown–Forsythe and Welch ANOVA. A total of 200 cells were analyzed per treatment (*n* = 200).

**Figure 2:**
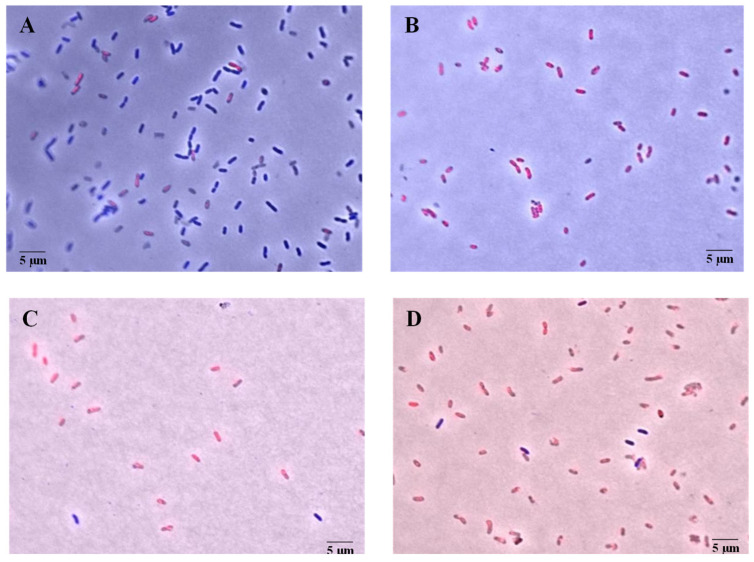
Fluorescence micrographs obtained during the live and dead assay with *X. citri* subsp. *citri* cells after 15 and 30 min of contact with the synergistic combination of 0.075% (*v*/*v*) citronella essential oil and 0.002% (*v*/*v*) rhamnolipid. (**A**): Negative control after 30 min; (**B**): Positive control after 30 min; (**C**,**D**): Cells treated with the combination of citronella essential oil and rhamnolipid after 15 and 30 min, respectively. Cells with permeabilized membranes are stained red, while cells with intact membranes are stained blue. Scale bar: 5 µm. Magnification of ×100.

After obtaining the synergistic combinations, a live and dead microscopy assay was performed to evaluate their mechanism of action against *X. citri* subsp. *citri*. For this assay, the synergistic combination with the lowest concentrations of both components was selected: 0.075% (*v*/*v*) citronella essential oil and 0.002% (*v*/*v*) rhamnolipid. The microscopic results are shown in Figure 1 and Figure 2. In Figure 1, after 15 min of treatment, more than 60% of the cells show permeabilized membranes, a percentage that increases to 80% after 30 min. In Figure 2, the fluorescence micrographs show that the staining of bacterial cells treated with the synergistic combination is similar to that of the positive control, indicating an effect on cell permeabilization.

### 3.5. Effect of Synergic Combination on Biofilm Production

**Figure 3:**
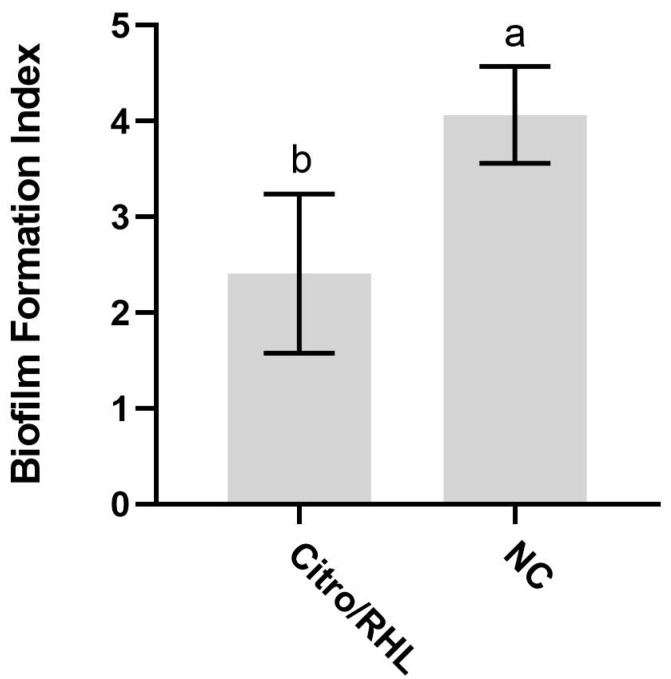
Biofilm formation index by *X. citri* subsp. *citri* bacteria in the presence of the synergistic combination of citronella essential oil and rhamnolipid. Biofilm formation index (absorbance: 590 nm/O.D. 600 nm). Negative control (NC). Treatments with citronella essential oil (EO) and rhamnolipid; concentration of 0.075% citronella and 0.002% RHL (*v*/*v*) (Citrol/RHL). Error bars represent the standard deviation of the mean. Letters (a) and (b) represent statistically similar groups (*p*-value > 0.05) according to the unpaired *t*-test and Welch’s ANOVA.

This assay was used to evaluate whether synergistic combinations act as an anti-biofilm by reducing virulence expression and biofilm formation [41,42]. The results of the biofilm assay shown in Figure 3 indicate that the synergistic concentrations of citronella oil 0.075% (*v*/*v*) and rhamnolipid 0.002% (*v*/*v*) evaluated are effective in reducing bacterial biofilm formation.

### 3.6. Time-Kill Assay

**Figure 4:**
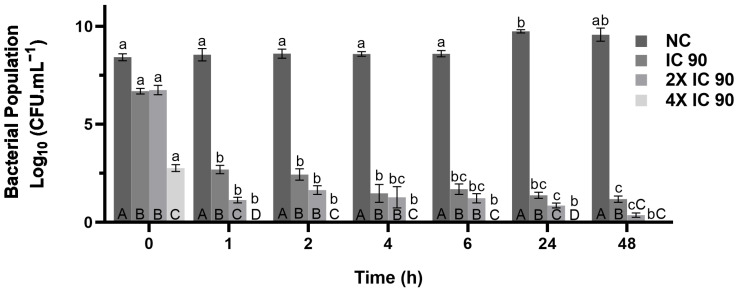
Time-kill kinetics of *X. citri* subsp. *citri* bacteria in the presence of the synergistic combination of citronella essential oil and rhamnolipid. IC 90 concentration of 0.075% citronella and 0.002% RHL (*v*/*v*). NC represents the negative control. Error bars represent the standard deviation of the mean. All the data are presented as the mean ± SD of independent experiments. Capital letters (e.g., A, B, C and D indicate statistical similarities between treatments within a time point; lower-case letters (e.g., a, b and c) indicate statistical similarities in a treatment across time points.

The time-kill assay was used to analyze the elimination rate of the components of the synergistic combination at a concentration of 0.075% (*v*, *v*) citronella essential oil and 0.002% (*v*, *v*) rhamnolipid, using the IC 90, 2× IC 90 and 4× IC 90 values show in Figure 4.

The synergistic combination reduced bacterial growth. Both the time (hours) and concentration variables (IC90, 2× IC90 and 4× IC90) had a significant effect on the number of cells recovered after exposure to the compound. The applied concentration was the dominant factor, accounting for 83% of the variation observed, while the hours of exposure accounted for 10% of the variation. At 1× IC 90, there was a bactericidal reduction of 5.55 log_10_ CFU/mL after 1 h compared to the negative control. The 4× the IC90 treatments differed from any other treatment in all time periods, except for cells treated with 2× IC90 at 48 h, where no cells were recovered. After 1 h of exposure, no cells were recovered when treated with 4× the IC90. All concentrations showed significant differences in the number of cells recovered compared to the controls at all time points.

## 4. Discussion

The results demonstrate the antimicrobial activity of rhamnolipid and the four essential oils against *X. citri* subsp. *citri*. Both the essential oils and rhamnolipid exhibited low inhibitory concentration 90% and minimal bactericidal concentration values, reinforcing their antimicrobial efficacy.

Previous studies indicate that essential oils such as palmarosa, clove, and geranium have an IC 90 of 1% against *Bacillus subtilis*, a Gram-positive bacterium [43]. However, in the present study, the same oils were even more effective against *X. citri* subsp. *citri*, achieving 90% inhibition at only 0.006%. This performance highlights the exceptional antimicrobial potency of these oils at extremely low concentrations. Similarly, citronella oil showed an IC 90 of 0.15% against *X. citri* subsp. *citri,* and Cerri and Esmerino reported an IC 90 of 0.1% against *S. aureus*, *Escherichia coli*, and *C. albicans* [44]. These data suggest that citronella has a broad spectrum of antimicrobial activity, although there are variations in the concentrations that can inhibit different species.

The chemical composition of the essential oils may explain these differences in antimicrobial activity. Palmarosa essential oil contains geraniol and neryl acetate as its major compounds (Table 2). Geraniol, a monoterpene with well-documented antimicrobial activity, disrupts the integrity of the bacterial cell membrane, increasing its permeability and leading to cell lysis [45]. Neryl acetate, although less studied, also as antimicrobial activity and may potentiate the effect of geraniol [46].

Geranium oil contains citronellol and geraniol. Citronellol, like geraniol, is a monoterpene with antimicrobial properties often associated with bacterial cell membrane disruption [47]. The synergy between these compounds may enhance their effectiveness, further disrupting the cell membrane and inhibiting microbial growth [48].

Clove essential oil presented chavibetol and eugenol acetate as major compounds. Chavibetol, a phenylpropanoid, showed antimicrobial activity, mainly against Gram-positive bacteria, by inhibiting essential enzymes and compromising the integrity of the cell membrane [49]. Eugenol acetate, a derivative of eugenol, is also widely recognized for its ability to destabilize cell membranes and inhibit enzymes critical for microbial metabolism [50].

Finally, citronella oil, which is composed primarily of geraniol and citral, stands out for its antimicrobial potential. Citral is known for its high antibacterial activity, and geraniol enhances antibacterial activity, especially when combined with other antimicrobial agents. Studies indicate that essential oils rich in citral and geraniol have activity against *X. citri* subsp. *citri*, with citral being the component with the greatest antibacterial efficacy among those tested [36].

Rhamnolipid showed an IC 90 of 0.3% against *X. citri* subsp. *citri*, which is consistent with the values reported for other microorganisms, including the Gram-positive bacteria *S. aureus*, *Bacillus cereus*; the Gram-negative bacteria *Enterobacter cloacae*, *Proteus mirabilis*; and the fungi *C. albicans*, *Aspergillus niger* [51]. These results underscore the efficacy of essential oils and rhamnolipid as viable and sustainable alternatives for the control of *X. citri* subsp. *citri*.

After determining the antibacterial concentration of each essential oil and rhamnolipid, the checkerboard assay was performed to evaluate the synergistic effect between the two compounds. From the results, the FICI was calculated and the synergistic effects were identified only for concentrations that resulted in values equal to or less than 0.5 [52]. Citronella essential oil, in combination with rhamnolipid, stood out with three synergistic interactions, as evidenced by a reduction in the 90% inhibitory concentration of both compounds when used together. The IC 90 of citronella essential oil was reduced by half, reaching 0.075% (*v*/*v*), while rhamnolipid showed even more pronounced reductions, with IC 90s of 0.009%, 0.004%, and 0.002% (*v*/*v*).

For the other essential oils with rhamnolipids, there was no synergy, but presented an additive effect, or independent effects (0.5 < ICIF ≤ 1), characterized by results equal to the sum of the individual effects of the compounds. This indicates that, although there is no potentiation, the compounds act independently, without antagonism [53].

Haba et al. [28] found that rhamnolipid-based emulsions increased the availability and antimicrobial efficacy of essential oils extracted from *Melaleuca alternifolia*, *Cinnamomum verum*, *Origanum compactum*, and *Lavandula angustifolia*, against *C. albicans* and methicillin-resistant *Staphylococcus aureus* (MRSA). These results support the potential of rhamnolipids to enhance the antimicrobial properties of essential oils.

However, studies investigating the synergistic combination of essential oils with rhamnolipids, especially against *X. citri* subsp. *citri*, are extremely scarce in the literature. Most of the available research focuses on associations between essential oils and antibacterials or antibiotics and other chemical substances. In this context, the data obtained in this project stand out as original and innovative, filling an important gap in the scientific field.

The results presented in Figure 1 and Figure 2 indicate a significant membrane-disrupting effect resulting from the combined application of citronella essential oil and rhamnolipid. The uptake of propidium iodide by the treated cells—indicated by the reddish fluorescence—indicates a loss of membrane integrity, a hallmark of bacterial cell death. Notably, as shown in Figure 3, over 60% of the cells exhibited permeabilized membranes within 15 min of treatment, with this percentage increasing to approximately 80% after 30 min. This time-dependent increase in membrane permeabilization reinforces the bactericidal potential of the formulation and highlights the importance of exposure time in enhancing antimicrobial efficacy.

The efficacy of citronella essential oil may be attributed, at least in part, to its major constituents, such as geraniol and citral. Previous studies have reported that citral disrupts the bacterial cell envelope and alters cytoplasmic density, thereby compromising structural and functional cellular integrity [36]. In this context, the observed effects may be explained by a synergistic interaction between the membrane-solubilizing properties of rhamnolipids and the structure-disrupting effects of citral, resulting in increased membrane permeability and, consequently, increased bacterial susceptibility.

Marin et al. [20] found that essential oils, especially from the *Cymbopogon* species, have antimicrobial activity against *X. citri* subsp. *citri*. Studies have shown that the active compounds present in EOs promote the disruption of the integrity of the bacterial cell membrane. Components such as terpenoids and phenols can interact with membrane lipids, increasing their permeability. This action results in the loss of ions, amino acids, and other essential metabolites, leading to cell death [20]. For example, the essential oil of *Amomum villosum* à Lour affected the membrane of *S. aureus*, resulting in the leakage of intracellular substances [54]. Studies also show that rhamnolipids have antibacterial properties when they interact with bacterial cell membranes, disrupting the cell membrane and leading to increased membrane permeability [55].

The results of the biofilm assay show that the evaluated synergistic concentrations of citronella oil 0.075% (*v*/*v*) and rhamnolipid 0.002% (*v*/*v*) have an effect on reducing bacterial biofilm formation. This reduction was greater than the growth shown in the negative control, indicating that the combination of compounds enhanced the antimicrobial activity by directly interfering with the mechanisms involved in biofilm formation and maintenance.

The essential oil of *C. nardus* (citronella) and its major component, geraniol, have been shown in the literature to inhibit biofilm formation and reduce *S. aureus* biofilm biomass. This antibiofilm activity is critical for preventing chronic infections and increasing the efficacy of antibacterial treatments [56]. In studies comparing citronella oil to commercial mouthwashes, citronella demonstrated superior efficacy in inhibiting the biofilm formation of *S. aureus* and *C. albicans*. Citronella oil resulted in greater microbial reduction and exhibited less cytotoxic effects compared to commercial solutions, making it a promising alternative for biofilm control [57]. The interaction between rhamnolipids and biofilm layers involves changes in surface free energy and surface charge. Rhamnolipids reduce the negative charge of the surface by removing negatively charged substances, such as humic-like substances, proteins, and fulvic acids. This leads to a reduction in the concentrations of carbohydrates and proteins in the EPS, which are essential for biofilm integrity [58].

In the time-kill assay (Figure 4), the exposure of *X. citri* subsp. *citri* to IC 90 resulted in a reduction in the bacterial population to less than half of the initial value within one hour. Note that increasing the concentration to 4× IC 90 accelerated bacterial elimination, reaching zero contamination within 1 hour and maintaining it for 48 h. Studies have also reported a similar effect, with citral and geraniol compounds in citronella oil eradicating *X. citri* subsp. *citri* within 30 min [36]. Thus, the results for IC 90, 2× IC 90 and 4× IC 90 after 1 h of contact confirm bactericidal activity, defined as a reduction of ≥3 log_10_ CFU/mL (≥99.9%), while bacteriostatic activity corresponds to a reduction of <3 log_10_ CFU/mL [59,60,61].

Despite the promising in vitro antibacterial activity of the combination of rhamnolipid and citronella essential oil against *X. citri* subsp. *citri*, translating these findings into large-scale agricultural applications presents significant challenges. While these natural compounds offer an environmentally sound alternative to traditional copper-based bactericides, their economic viability remains uncertain [11].

The commercial feasibility of biosurfactants and essential oils in agriculture depends on several factors, including production cost, formulation stability, and shelf life [62]. For example, rhamnolipids, while effective, require optimized fermentation processes to reduce costs [63], while essential oils are limited by volatility and degradation under field conditions [62]. In addition, synergistic interactions that reduce the required concentration of active compounds need to be confirmed in real-world settings, where environmental factors can reduce efficacy.

Furthermore, the adoption of biobased alternatives in citrus canker management programs depends not only on efficacy, but also on cost competitiveness. Small-scale laboratory successes do not always translate into economically viable large-scale applications, as has been seen with other biocontrol agents [64]. Therefore, future research should prioritize field trials that evaluate long-term performance, formulation stability, and cost-effectiveness to determine whether rhamnolipid–essential oil combinations can be integrated into sustainable citrus production systems.

## 5. Conclusions

This study demonstrates the potential of essential oils and rhamnolipids as sustainable alternatives for the control of citrus canker, caused by *X. citri* subsp. *citri*. The evaluated EOs, citronella, palmarosa, geranium, and clove, showed antibacterial activity against *X. citri* subsp. *citri*. Rhamnolipids also showed antimicrobial activity, reinforcing their role as a viable alternative to traditional copper-based treatments. The synergistic combination of citronella oil with rhamnolipids was particularly effective, as this synergistic mixture not only permeabilized bacterial cell membranes, but also inhibited biofilm formation, a critical factor in bacterial virulence and persistence. Of particular note was highlighting the time required to eliminate the bacterial population rate, resulting in total death within a few hours.

Therefore, the results of this study provide a basis for the development of sustainable antimicrobial formulations for agricultural use. By reducing the reliance on copper-based products that pose environmental and health risks, the use of EOs and rhamnolipids offers a safer and more environmentally friendly treatment approach to disease control.

## Figures and Tables

**Table 1:** Inhibitory concentrations 90% (IC 90) and minimum bactericidal concentrations (MBCs) for samples against *X. citri* subsp. *citri* in % (*v*/*v*).

Samples	IC 90	MBC
Palmarosa	0.06	0.06
Citronella	0.15	0.25
Geranium	0.06	0.06
Clove	0.06	0.06
Rhamnolipids	0.3	0.5

**Table 2:** Results obtained by GC/MS chromatographic analysis for palmarosa oil.

Compounds	Ret. Time	Height %	Area %
β-Myrcene	4.282	1.65	1.08
β-cis-Ocimene	5.142	3.56	2.46
Linalool	6.057	4.75	3.02
Geraniol	9.258	61.09	71.2
Citral	9.611	1.68	1.16
Neryl acetate (Z)	11.999	24.84	19.17
Caryophyllene	13.063	2.43	1.91
Total			100

**Table 3:** Results obtained by GC/MS chromatographic analysis for citronella oil.

Compounds	Ret. Time	Height %	Area %
Sulcatone	4.211	2.00	0.99
Myrcene	4.284	1.94	0.59
D-Limonene	4.927	2.45	3.61
Linalol	6.057	2.04	1.79
Citronellal	7.099	2.32	7.78
Cis-2-Decen-1-ol	8.156	2.08	0.59
Citronellol	8.625	2.23	6.33
Citral (Z)	8.992	2.43	9.48
Geraniol	9.239	3.16	43.68
Citral (E)	9.614	2.46	13.53
Citronellyl acetate	11.315	2.30	1.75
Neryl Acetate (Z)	11.992	2.36	4.74
Caryophyllene	13.062	2.61	3.42
Γ-Muurolene	15.034	2.59	1.72
Total			100

**Table 4:** Results obtained by GC/MS chromatographic analysis for geranium oil.

Compounds	Ret. Time	Height %	Area %
Linalool	6.060	6.46	5.2
Rose Oxide (E)	6.661	0.66	0.54
(+)-Isomenthone (Cis)	7.253	4.75	4.19
(+)-Isomenthone (Trans)	7.481	4.65	4.28
Citronellol	8.643	31.52	34.63
Citral	8.994	0.6	0.51
Geraniol (Z)	9.218	16.48	15.73
Citronellyl formate	9.648	10.97	11.02
Geranyl Bromide	10.247	3.75	3.48
2,6-Octadiene, 2,6 dimethyl	11.319	0.62	0.53
α-Copaene	12.056	0.7	0.61
β-Bourbonene	12.285	1.58	1.48
Caryophyllene	13.066	1.32	1.26
Citronellyl propionate	13.271	0.81	0.77
β-Bisabolene	13.949	0.93	0.95
Citronellyl butyrate	15.073	0.82	0.66
γ-Muurolene	15.190	1.53	1.87
Geranyl isobutyrate	15.748	1.01	0.9
Phenylethyl tiglate	16.420	1.32	1.34
epi-γ-Eudesmol	17.307	6.5	7.33
Geranyl tiglate	18.589	1.37	1.31
Total			100

**Table 5:** Results obtained by GC/MS chromatographic analysis for clove oil.

Compounds	Ret. Time	Height %	Area %
Chavibetol	11.617	58.99	63.84
Caryophyllene	13.071	6.64	5.3
Eugenol Acetate	15.175	34.37	30.86
Total			100

**Table 6:** Fractional Inhibitory Concentration Index (FICI) of the combination between essential oils and rhamnolipid against *X. citri* subsp. *citri*, in % (*v*/*v*).

Essential Oils	RHL *							
	0.002	0.004	0.009	0.018	0.030	0.075	0.15	0.3
**Citronella**								
**0.15**	1	1	1	1	1	1	1	2
**0.075**	0.5	0.5	0.5	0.6	0.6	0.7	1	1.5
**0.03**							0.7	1.2
**0.018**							0.6	1.1
**Palmarosa**								
**0.06**	1	1	1	1	1	1	1	2
**0.03**							1	1.5
**0.015**							0.7	1.2
**0.0075**							0.6	1.1
**Geranium**								
**0.06**	1	1	1	1	1	1	1	2
**0.03**							1	1.5
**0.015**								1.2
**0.0075**								1.1
**Clove**								
**0.06**	1	1	1	1	1	1	1	2
**0.03**							1	1.5
**0.015**								1.2
**0.0075**								1.1

* Rhamnolipid = RHL.

## Data Availability

The original contributions presented in this study are included in the article. Further inquiries can be directed to the corresponding author.
